# Intracellular accumulation of aggregated pyroglutamate amyloid beta: convergence of aging and Aβ pathology at the lysosome

**DOI:** 10.1007/s11357-012-9403-0

**Published:** 2012-04-04

**Authors:** Line De Kimpe, Elise S. van Haastert, Archontia Kaminari, Rob Zwart, Helma Rutjes, Jeroen J. M. Hoozemans, Wiep Scheper

**Affiliations:** 1 Department of Genome Analysis, Academic Medical Center, Meibergdreef 9, 1105 AZ Amsterdam, The Netherlands; 2 Department of Pathology, VU University Medical Center, De Boelelaan 1117, 1081 HV Amsterdam, The Netherlands; 3 Hycult Biotech, Frontstraat 2a, 5405 PB Uden, The Netherlands; 4 Department of Neurology, Academic Medical Center, Meibergdreef 9, 1105 AZ Amsterdam, The Netherlands; 5 Department of Genome Analysis, Academic Medical Center, P.O. Box 22660, 1100 DE Amsterdam, The Netherlands

**Keywords:** Alzheimer’s disease, Pyroglutamate amyloid beta, Lysosomal pathology

## Abstract

Deposition of aggregated amyloid beta (Aβ) is a major hallmark of Alzheimer’s disease (AD)—a common age-related neurodegenerative disorder. Typically, Aβ is generated as a peptide of varying lengths. However, a major fraction of Aβ peptides in the brains of AD patients has undergone posttranslational modifications, which often radically change the properties of the peptides. Aβ_3(pE)-42_ is an N-truncated, pyroglutamate-modified variant that is abundantly present in AD brain and was suggested to play a role early in the pathogenesis. Here we show that intracellular accumulation of oligomeric aggregates of Aβ_3(pE)-42_ results in loss of lysosomal integrity. Using a novel antibody specific for aggregates of AβpE3, we show that in postmortem human brain tissue, aggregated AβpE3 is predominantly found in the lysosomes of both neurons and glial cells. Our data further demonstrate that AβpE3 is relatively resistant to lysosomal degradation, which may explain its accumulation in the lysosomes. The intracellular AβpE3 aggregates increase in an age-dependent manner. The results presented in this study support a model where Aβ pathology and aging converge, leading to accumulation of the degradation-resistant pE-modified Aβ in the lysosomes, lysosomal dysfunction, and neurodegeneration.

**Electronic supplementary material:**

The online version of this article (doi:10.1007/s11357-012-9403-0) contains supplementary material, which is available to authorized users.

## Introduction

Alzheimer’s disease (AD) is a common progressive neurodegenerative disorder of which the prevalence is steeply increasing in the aging society. Neuropathologically, AD is characterized by deposits of aggregated proteins: intracellular aggregates of hyperphosphorylated tau in neurofibrillary tangles and extracellular β-amyloid (Aβ) in plaques. According to the widely supported amyloid cascade hypothesis, increased levels and the formation of assemblies of Aβ is one of earliest events in AD pathogenesis (Hardy and Selkoe 2002). Indeed, Aβ is prone to self-aggregate and the formation of the resulting aggregates is strongly linked to the development of the disease. However, despite enormous research efforts, the exact site and mechanism of Aβ toxicity is still not entirely elucidated. A major obstacle in Aβ research is that Aβ is not a single entity but a peptide with great molecular heterogeneity (De Kimpe and Scheper 2010).

Aβ is generated as a peptide of varying lengths by combined action of β- and γ-secretase through proteolytic processing of the amyloid precursor protein (APP). Previously extracellular fibrillar Aβ as found in the plaques was considered to be the main toxic species. However, recently it has become apparent that soluble oligomeric Aβ aggregates and intracellular Aβ play important roles in the pathogenesis of AD (Haass and Selkoe 2007).

In addition to the full-length Aβ_1-x_ peptides, various modified Aβ variants can be detected in the human brain (Saido et al*.* 1996). These variants are truncated at their N- or C-termini and/or harbor different posttranslational modifications resulting in the generation of Aβ peptides with different physical and chemical properties. An abundant Aβ variant in human brain is the pyroglutamate (pE)-modified AβpE3. This N-terminal pE modification of Aβ can be catalyzed by the enzyme glutaminyl cyclase (QC) (Schilling et al*.* 2004). AβpE3 peptides show a higher stability (Kuo et al*.* 1998), increased aggregation propensity (Schilling et al*.* 2006), and neurotoxicity (Russo et al*.* 2002; Youssef et al*.* 2008) compared to the full-length unmodified Aβ. A mouse model selectively expressing this variant shows that intraneuronal accumulation of Aβ_3(pE)-42_ is sufficient for triggering neuronal loss and behavioral deficits (Wirths et al*.* 2009). This indicates that Aβ_3(pE)-42_ is a pathogenic species relevant to the progression of the disease. Because of its aggregation ability, AβpE3 has been suggested to provide the “seed” for the Aβ aggregation in AD (Schilling et al. 2006). In support of this, prophylactic treatment with a QC inhibitor results in decreased Aβ_x-40/42_ levels, reduced plaque burden, diminished gliosis, and improved spatial learning, as shown in two different APP transgenic mouse models (Schilling et al*.* 2008). Therefore, the pE-modified Aβ peptides are a potential target for the treatment of the disease. However, the precise role of pE-modified Aβ in the pathogenesis of the disease is still elusive. In this study, we focused on the pathogenic involvement of non-fibrillar aggregates of Aβ_3(pE)-42_. Previously, our group demonstrated that oligomeric Aβ_1-42_ is rapidly internalized by neuronal cells to ultimately accumulate in the lysosomal compartment. Inhibition of uptake of oligomeric Aβ_1-42_ decreases toxicity (Chafekar et al*.* 2008) suggesting that the lysosome is involved in Aβ toxicity. In addition, dysfunction of the endo-/lysosomal pathway is an early phenomenon in AD pathology. From previous studies, it is know that lysosomal damage plays a role in soluble Aβ_1-42_-mediated cell death (Ditaranto et al. 2001). Lysosomal dysfunction plays an important role in the disease (Nixon et al. 2008), but it is not known whether AβpE3 affects the lysosomes. It is of interest to investigate the mode of toxicity of this specifically modified Aβ form because its formation can potentially be prevented using a QC inhibitor. Therefore, in this study, we investigated the involvement of the lysosome in the pathomechanistic role of AβpE3 aggregates. Using cell models, in vitro lysosomal degradation assays and analysis of postmortem human brain material, we obtained data that support a model showing convergence of AβpE3 pathology and aging at the lysosome.

## Results

### Aβ_3(pE)-42_ oligomers cause lysosomal membrane permeabilization

**Fig. 1:**
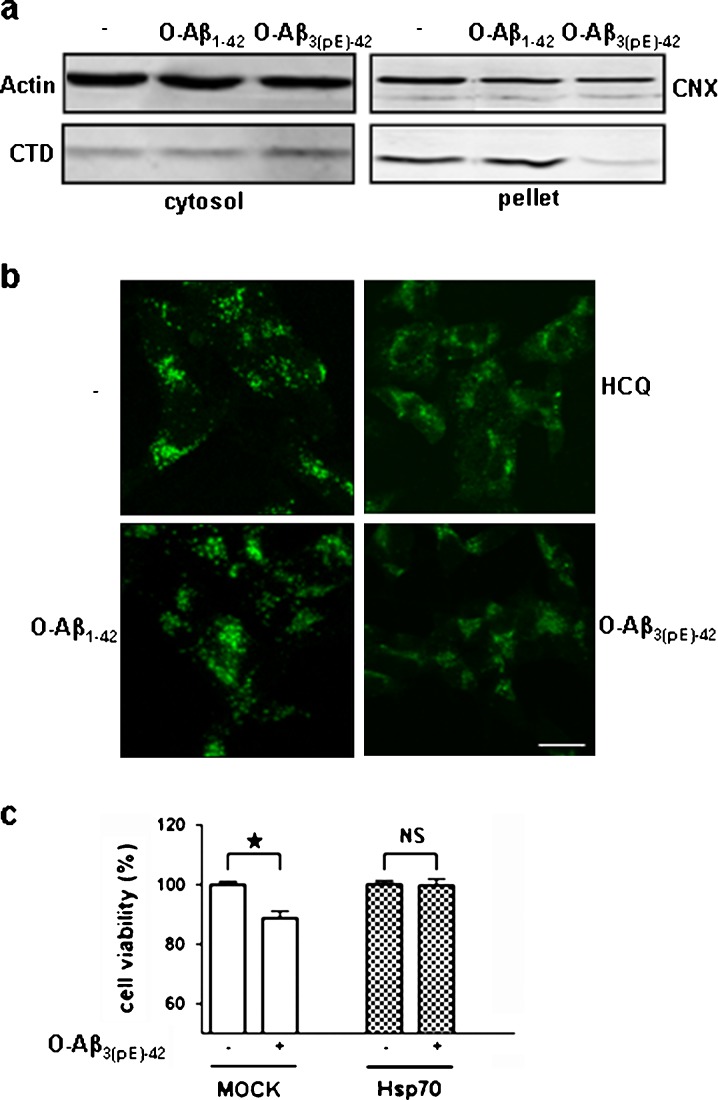
Aβ_3(pE)-42_ oligomers cause LMP. **a** Western blot analysis showing the protein levels of CTD in the cytosolic and in the pellet fraction of untreated (*minus sign*) cells and after treatment with 1 μM Αβ_1-42_ or Αβ_3(pE)-42_ oligomers for 16 h. β-actin and calnexin are depicted as protein loading control. The experiment was performed three times and representative blots are shown. **b** Confocal pictures of differentiated SK-N-SH cells after immunofluorescent staining of CTD on untreated (*minus sign*), HCQ-, Αβ_1-42_-, and Aβ_3(pE)-42_-treated cells. **c** Mock-transfected- or Hsp70-transfected HeLa cells were incubated in the absence (*minus sign*) or presence (*plus sign*) of 2 μM Αβ_1-42_ or Αβ_3(pE)-42_ oligomers for 24 h. Cell viability was measured using an MTT assay and is depicted as a percentage of the untreated (*minus sign*) cells. The graph represents the mean ± SEM (*n* = 16) from four independent experiments. *p < 0.01 as calculated by an unpaired two-tailed Student's *t* test

To investigate whether Aβ_3(pE)-42_ oligomers affect the integrity of the lysosomes, lysosomal membrane permeabilization (LMP) was analyzed in differentiated SK-N-SH cells. A distinctive sign of LMP is the translocation of soluble lysosomal components, from the lysosomal lumen to the cytosol (Boya et al*.* 2003). Differentiated SK-N-SH cells were treated with Aβ oligomers and the leakage of cathepsin D (CTD), a soluble lysosomal enzyme, from the lysosomal lumen to the cytosol was analyzed after subcellular fractionation on Western blot and by immunofluorescent staining. Treatment with Aβ_3(pE)-42_ oligomers led to a decrease of CTD in the membrane fraction (which also contained the lysosomes) whereas the cytosolic CTD was increased. However, treatment with Aβ_1-42_ oligomers did not change the levels of CTD neither in the membrane fraction, nor in the cytosolic fraction (Fig. 1a). The result was confirmed by immunofluorescent staining of CTD. Untreated cells reveal a particulate staining pattern, indicative of its localization within lysosomes (Fig. 1b). After treatment with Aβ_3(pE)-42_ oligomers, the staining was less punctuate and bright but more diffusely distributed throughout the entire cytoplasm, indicating the enzyme had been released from the lysosomes to the cytoplasm. Treatment with hydroxychloroquine (HCQ), an established inducer of LMP, gave a similar result as the cells treated with the Aβ_3(pE)-42_ oligomers. Treatment with Aβ_1-42_ oligomers shows the same pattern but to a lesser extent.

Taken together these data demonstrate that Aβ_3(pE)-42_ oligomers are more potent inducers of LMP than Αβ_1-42_ oligomers. LMP is toxic for cells because it leads to impaired lysosomal function and aberrant exposure of cellular components to lysosomal enzymes. Since Hsp70 has been reported to stabilize lysosomes and thereby inhibit LMP (Kirkegaard et al*.* 2010), we investigated if increased levels of Hsp70 could inhibit Aβ_3(pE)-42_ oligomer toxicity. Therefore mock- or Hsp70-transfected HeLa cells were treated with 2 μM Aβ_3(pE)-42_ oligomers. Using the MTT cell viability assay, the effect of Hsp70 overexpression on Aβ toxicity was analyzed. Overexpression of Hsp70 completely inhibited Aβ_3(pE)-42_ oligomer toxicity (Fig. 1c). These data indicate that disruption of lysosomal function contributes to Aβ_3(pE)-42_ oligomer toxicity.

### Detection of aggregated AβpE3 in postmortem human brain tissue

**Fig. 2:**
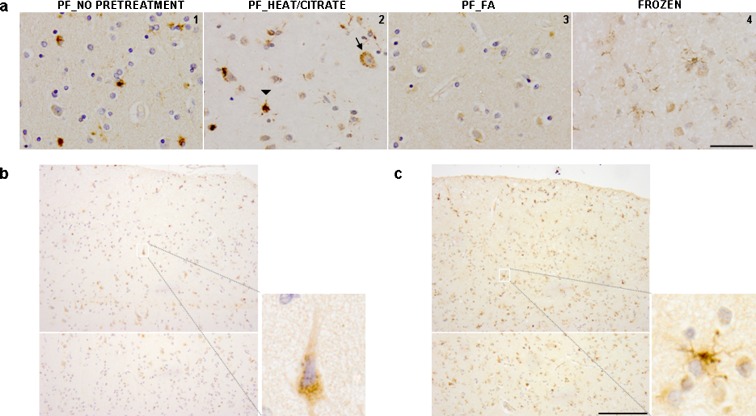
Detection of aggregated AβpE3 in postmortem human brain tissue. **a** Paraffin-embedded (*1–3*) or frozen (*4*) sequential sections from temporal cortex of an AD patient were stained with the O-AβpE3 antibody. For the paraffin-embedded sections (*PF*), different protocols were compared: without antigen retrieval (*1*) or with antigen retrieval: 10 min microwave heating in a citric acid buffer pH 6 (*2*) or 10-min formic acid (*FA*) pretreatment (*3*). *Arrow* depicts a neuron and *arrowhead* indicates a glial cell. Scale bar (1–4), 50 μm. **b** Paraffin-embedded heat-treated sections from temporal cortex of an AD patient stained with the O-AβpE3 antibody. *Inset* shows a higher magnification picture of a neuron. **c** Paraffin-embedded heat-treated sections from temporal cortex of an AD patient stained with the O-AβpE3 antibody. *Inset* shows a higher magnification picture of a glial cell. Scale bar, 200 μm

**Fig. 3:**
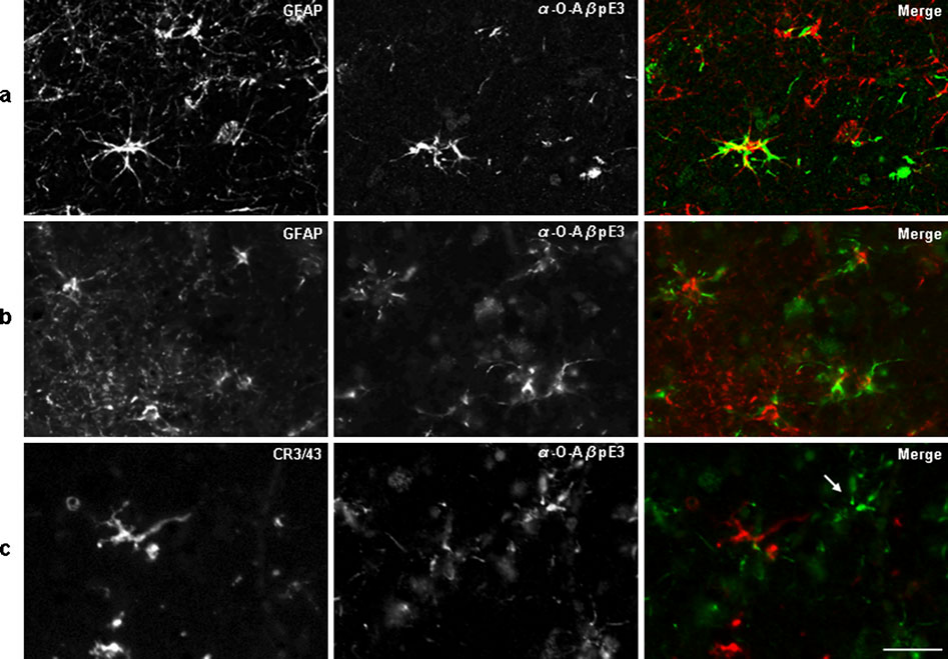
Intracellular AβpE3 aggregates are present in astrocytes in human brain. Epifluorescent microscopic images of double fluorescent immunohistochemistry stainings for AβpE3 oligomers and GFAP or CR3/43. Paraffin-embedded sections from temporal cortex of an AD patient were double stained with GFAP (*red*) as marker for astrocytes (**a**, **b**) or with CR3/43 (*red*) as a marker for microglia (**c**), and with the O-AβpE3 antibody (*green*). The *arrow* shows the typical star-like O-AβpE3 staining pattern associated with glial cell staining while CR3/43 staining is clearly absent. Scale bar, 25 μm

To investigate whether these in vitro observations reflect a process that is relevant in the human brain, we analyzed postmortem brain tissue of AD and control patients using an antibody against oligomers of Aβ_3(pE)-42_, the O-AβpE3. Extensive characterization showed that the O-AβpE3 detects a conformation-specific epitope because it detects oligomeric aggregates of Aβ_3(pE)-42_ but not monomeric Aβ_3(pE)-42_ (see Online resource Fig. S1). The epitope is enriched in early aggregates of Aβ_3(pE)-42_. O-AβpE3 does not detect Aβ_1-42_ or Aβ_3-42_ oligomers, showing that it is specific for pE3-modified Aβ oligomers (Online resource Fig. S1). Remarkably, in postmortem tissue of human brain material, the O-AβpE3 antibody does not recognize plaques but showed a strong intracellular staining pattern. This staining pattern is similar on formalin-fixed or frozen tissue sections but is nearly abolished by formic acid pretreatment, indicating that also in brain material the O-AβpE3 antibody detects a conformation-specific epitope (Fig. 2a). Aggregates of Aβ_3(pE)-42_ are structurally different than Aβ_1-42_ aggregates as indicated by differential recognition by different Aβ antibodies and TEM analysis (Online resource Fig. S2). This is corroborated by the analysis of co-aggregates of Aβ_3(pE)-42_ and Aβ_1-42_, which shows that the antibody only shows strong reactivity with (nearly) pure Aβ_3(pE)-42_ aggregates (Online resource Fig. S2). These specific properties may explain why the staining pattern is different from that obtained with widely used Aβ antibodies like 6E10. Based on the morphology of the cells, intracellular staining could be observed in neurons and glial cells. Neurons showed a diffuse and fine granular staining of the cell body, with no staining of the extensions, whereas the glial cells showed more compact granular staining of the cell body as well as of the extensions. The intraneuronal staining was found predominantly in the pyramidal neurons in layers III and V of the cortex (Fig. 2b), while the intraglial staining was spread throughout all the layers of the cerebral cortex (Fig. 2c). In order to identify which type of glial cells stain positive with the O-AβpE3 antibody, double immunohistochemistry (IHC) with microglia- (CR3/43) and astrocyte- (GFAP) specific markers was performed (Fig. 3). The double IHC of GFAP and the O-AβpE3 antibody showed two different types of co-localization. Either a strong co-localization of O-AβpE3 with GFAP was found (Fig. 3a) or O-AβpE3 staining was closely associated with GFAP staining (Fig. 3b). In contrast, no co-localization or association with CR3/43 was observed (Fig. 3c). Based on these findings, we conclude that the glial AβpE3 aggregates are mainly present in astrocytes and not in microglia.

### Aggregated AβpE3 is present in the lysosomes of postmortem human brain

**Fig. 4:**
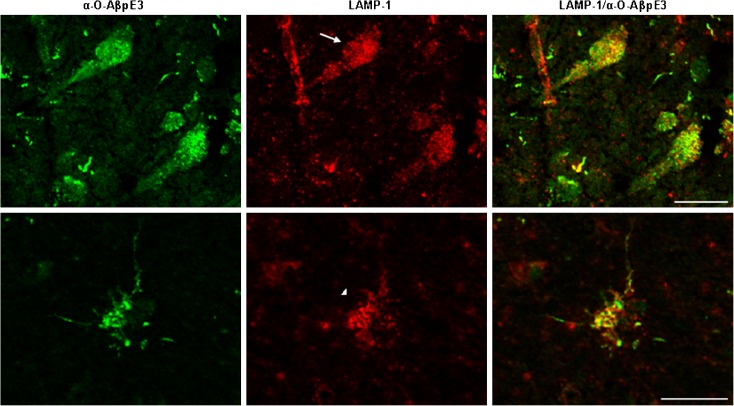
Aggregated AβpE3 is present in the lysosomes of *postmortem* human brain. Confocal microscopic images of frozen sections from temporal cortex of an AD patient double stained with the O-AβpE3 antibody (*green*) and with a LAMP-1 antibody (*red*). Co-localization is depicted in *yellow*. *Arrow* depicts a neuron and *arrowhead* indicates a glial cell. Scale bar, 25 μm

To investigate if the aggregated AβpE3 species observed in the neurons and glial cells of the postmortem brain tissue are also present in the lysosomal compartment, we performed double immunofluorescence. A clear co-localization of the lysosomal marker LAMP-1 and the O-AβpE3 antibody was observed in neurons as well as in glial cells (Fig. 4). Some LAMP-1-negative O-AβpE3 staining is observed; however, the majority of the staining co-localizes with LAMP-1. These data demonstrate that the AβpE3 aggregates accumulate in the lysosomal compartment.

### pE modification confers resistance to lysosomal degradation of Aβ

**Fig. 5:**
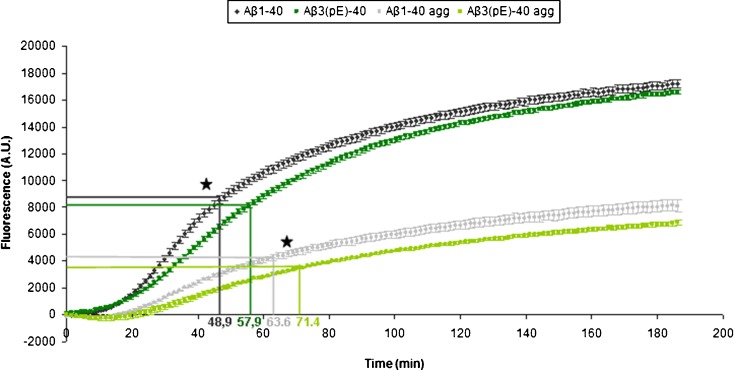
pE modification confers resistance to lysosomal degradation of Aβ. Internally quenched fluorescent Αβ_1-40_ and Aβ_3(pE)-40_ peptides (pre-aggregated or not) were used in a degradation assay employing recombinant CTD. Degradation of the quenched Aβ peptides is indicated as the accumulated fluorescence over time. The graph represents the mean ± SEM from two independent experiments (*n* = 10). *p < 0.05 as calculated by an unpaired two-tailed Student's *t* test

The accumulation of AβpE3 aggregates in the lysosomal compartment suggests that they are not efficiently degraded. Therefore, we investigated whether the pE-modified Aβ is more resistant to lysosomal degradation. To this end, a degradation assay with the major lysosomal hydrolase CTD was performed. For this purpose, we used internally quenched fluorescent Aβ peptides. Degradation of the peptides removes the quencher and restores the fluorescent signal (Reits et al*.* 2003), thus allowing analysis of degradation over time by monitoring the increase in fluorescence. Incubation of Aβ_1-40_ or Aβ_3(pE)-40_ with CTD showed a clear increase in fluorescence indicative of their degradation (Fig. 5). However, the increase in fluorescence observed for the Aβ_3(pE)-40_ is significantly slower than the increase observed for Aβ_1-40_. Indeed, the time to reach the half-max fluorescence of Aβ_1-40_ degradation is 48.9 min (*t*_1/2_ = 48.9 ± 4.3 min, mean ± SD) whereas the half-max fluorescence of Aβ_3(pE)-40_ degradation is reached at 57.9 min (*t*_1/2_ = 57.9 ± 3.1 min, mean ± SD). To investigate the effect of aggregation on degradation, we used pre-aggregated Aβ_1-40_ and Aβ_3(pE)-40_ in the CTD assay. We observed that due to aggregation the reaction is slower and the maximal fluorescence was strongly reduced. This effect is more pronounced for Aβ_3(pE)-40_ than for Aβ_1-40_ since the maximal fluorescence of the Aβ_3(pE)-40_ degradation is lower than the values reached for Aβ_1-40_ degradation. In addition, the half-max fluorescence of pre-aggregated Aβ_1-40_ degradation is reached at 63.6 min (*t*_1/2_ = 63.6 ± 10.0 min, mean ± SD) while the half-max fluorescence of pre-aggregated Aβ_3(pE)-40_ degradation at 71.4 min (*t*_1/2_ = 71.4 ± 2.24 min, mean ± SD). Upon aggregation, the overall degradation is hampered, but the observed difference between the peptides is retained. These results demonstrate that the pyroglutamate modification in combination with aggregation indeed confers resistance to proteolysis by CTD, which will facilitate their accumulation in the lysosomes.

### Intracellular AβpE3 aggregates increase with age

**Fig. 6:**
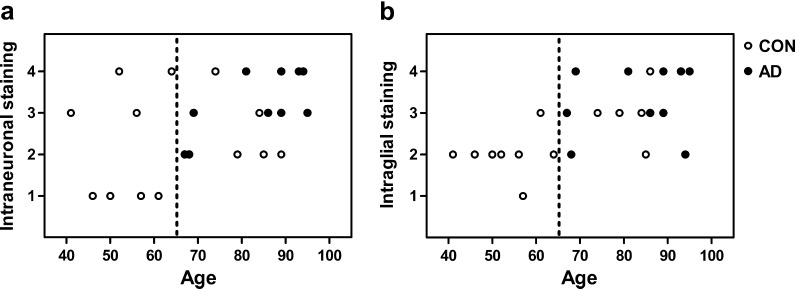
Intracellular-aggregated AβpE3 increases with the progression of AD pathology and age. Shown are two different scatter plots demonstrating the relation between age and intraneuronal (**a**) or age and intraglial staining are shown (**b**). Each point represents an individual patient where *closed circles* are diagnosed as AD and *open circles* are diagnosed as a control (*CON*). The corresponding correlation coefficients are described in the text

To obtain further insight in the involvement of aggregates of AβpE3 during the progression of AD, temporal cortex sections from 16 non-demented controls and 11 AD patients with different Braak scores for AD pathology were stained with O-AβpE3 (Table 1). The slides were analyzed using a qualitative grading system. The result of this analysis for all the stained slides was summarized in a table (Table 1) and in two scatter plots (Fig. 6). First, we performed nonparametric statistical analysis on the whole cohort confirming that the intraneuronal and the intraglial staining were two independent variables (*r* = 0.332, *p* = 0.09). Subsequently, it was tested whether either staining was correlated to patient data including gender, age, PMD, Braak stage, diagnosis, and ApoE genotype. For the intraneuronal staining, we only found a positive correlation with age (*r* = 0.442, *p* = 0.021). For the intraglial staining, a correlation with age (*r* = 0.628, *p* < 0.001), Braak stage (*r* = 0.524, *p* = 0.005), and diagnosis (*r* = 0.409, *p* = 0.034) was found. However, partial correlation analysis showed that after correction for age, intraglial staining was no longer significantly associated with Braak stage (*r* = 0.267, *p* = 0.187) or diagnosis (*r* = 0.232, *p* = 0.255). In contrast, after correction for Braak stage or diagnosis, intraglial staining remained significantly associated with age (*r* = 0.493, *p* = 0.01 and *r* = 0.564, *p* = 0.003, respectively). Taken together, this analysis showed that intraneuronal as well as intraglial staining increases with aging. Next, we analyzed the age dependence in relation to the diagnosis. Strikingly, in the control group, the intraneuronal staining did not correlate with age (*r* = 0.266, *p* = 0.319), whereas in the AD group a strong correlation (*r* = 0.697, *p* = 0.017) was still observed. This demonstrated that in the AD group intraneuronal staining increased with age. For the intraglial staining, the correlation remained strictly dependent on age. In the control group, the intraglial staining remained strongly correlated with age (*r* = 0.745, *p* = 0.001); however, within the AD group the correlation with age (*r* = 0.259, *p* = 0.443) was lost probably because in the AD group the range of age differences is smaller. In conclusion, these results indicate that intracellular accumulation of aggregated AβpE3 in glial cells is related to age and in neurons to a combination of age and AD pathology.

**Table 1:** *Postmortem* brain material used in this study

	Diagnosis	Gender	Age	PMD	Break stage	ApoE	Intraneuronal Aβ	Intraglial Aβ
1	AD	F	81	10:20	VI	33	4	4
2	AD	F	89	04:30	VI	43	4	3
3	AD	M	69	05:00	VI	43	3	4
4	AD	M	69	06:30	VI	33	2	2
5	AD	F	67	05:05	VI	32	2	3
6	AD	F	94	04:30	V	33	4	2
7	AD	M	93	04:30	V	43	4	4
8	AD	F	89	06:30	V	33	3	4
9	AD	M	67	10:00	V	33	2	3
10	AD	F	95	05:15	IV	33	3	4
11	AD	F	86	04:55	IV	43	3	3
12	CON	F	89	15:40	III	32	2	3
13	CON	M	86	05:35	III	43	3	4
14	CON	M	74	05:00	III	43	4	3
15	CON	F	93	07:05	II	33	4	4
16	CON	F	86	06:25	II	43	3	3
17	CON	F	85	04:40	II	43	2	2
18	CON	M	84	07:05	I	33	3	3
19	CON	M	79	06:30	I	33	2	3
20	CON	M	57	05:40	I	43	1	1
21	CON	M	50	05:30	I	33	1	2
22	CON	M	56	09:15	0	43	3	2
23	CON	F	41	10:30	0	43	3	2
24	CON	F	64	08:35	0	42	4	2
25	CON	F	52	06:50	0	33	4	2
26	CON	F	61	06:50	0	32	1	3
27	CON	F	46	10:25	0	33	1	2

Listed are diagnosis, gender, age (in years), *postmortem* delay (PMD, in hours: minutes), Braak stage, ApoE genotype, intraneuronal, and intraglial Aβ staining intensity scored by two independent observers

## Discussion

In the present study, we investigated the involvement of oligomeric aggregates of AβpE3 in the pathogenesis of AD. Previously, our group found that oligomeric Aβ_1-42_ is rapidly internalized by neuronal cells to ultimately accumulate in the lysosomal compartment. Inhibition of uptake of oligomeric Aβ_1-42_ decreases toxicity (Chafekar et al*.* 2008), suggesting that the lysosome is involved in Aβ toxicity. It was reported before that Aβ_1-42_ could cause LMP (Ditaranto et al*.* 2001). Using subcellular fractionation and immunofluorescence, we demonstrated that Aβ_3(pE)-42_ oligomers induce LMP more potently than Aβ_1-42_ oligomers. The relevance of Aβ_3(pE)-42_-induced LMP is indicated by our observation that inhibition of LMP by overexpression of Hsp70 provides protection against Aβ_3(pE)-42_ toxicity. We hypothesize that the observed increase in lysosomal damage after treatment with Aβ_3(pE)-42_ oligomers compared to oligomers of Aβ_1-42_ is facilitated by their increased resistance to lysosomal proteolysis, thereby allowing these aggregates to accumulate in the lysosomes. An in vitro lysosomal degradation assay showed that the pE modification as well as its consequent increased aggregation propensity hampers the degradation of Aβ_3(pE)-40_ by CTD at lysosomal pH (Fig. 5).

To further investigate the role of AβpE3 aggregates in the human brain, a novel antibody was generated against oligomeric Aβ_3(pE)-42_. The antibody is specific for aggregated 3pE-modified Aβ because it does not detect Aβ_3(pE)-42_ monomers or oligomeric Aβ_3-42_ or Aβ_1-42_. It is likely that the antibody detects 3pE-modified Aβ of different lengths, as the N-terminus appears as the major determinant of the structure of the aggregates. The addition of two N-terminal residues (Aβ_1-42_) or omission of the N-terminal pE modification (Aβ_3-42_) destroys the conformational epitope (Online resource Fig. S1). This makes it unlikely that aggregates of N-terminal variants like AβpE11 will be recognized by the antibody. In human postmortem brain tissue, intracellular-aggregated AβpE3 was detected in neurons as well as in glial cells. Interestingly, the O-AβpE3 does not recognize plaques, whereas it was previously shown that an Aβ_1-42_ oligomer-specific antibody did show plaque binding (Kayed et al*.* 2003). Recently, a different AβpE3 oligomer antibody was reported that also predominantly detects intracellular AβpE3 in human hippocampus and frontal cortex (Wirths et al*.* 2010). Analysis of co-aggregates of Aβ_3(pE)-42_ and Aβ_1-42_ (Online resource Fig. S2c) demonstrates that O-AβpE3 only detects (nearly) pure pE aggregates. The levels of these specific aggregates may be low compared to the massive Aβ deposits in the plaques and therefore not detected by regular Aβ staining protocols. In addition, it is possible that these aggregates are predominantly present intracellularly, or that the structural epitope is not present or accessible due to different structure of the AβpE3 in extracellular aggregates. Evidence from transgenic mouse models suggests that the intracellular accumulation of Aβ precedes plaque formation and may play an important role in neurodegeneration (Wirths et al*.* 2001). Because Aβ peptides are being produced in various cellular compartments of both neurons and glial cells (Hoozemans et al*.* 2006), it is not surprising that Aβ is found intracellularly. Moreover, in human primary neurons Aβ oligomers have been shown to be produced intracellularly as well, indicating that aggregation can occur inside the cell (Walsh et al*.* 2000). A second mechanism that may contribute to the intracellular accumulation is reuptake of Aβ peptides from the extracellular space (Chafekar et al*.* 2008; Nielsen et al*.* 2010; Nielsen et al*.* 2009). The uptake of Aβ by astrocytes can be attributed either to their phagocytic capacity (Watabe et al*.* 1989) or to a receptor-mediated process. Interestingly, receptor for advanced glycation end products (RAGE)-Aβ complexes has been shown to be internalized and co-localize with the lysosomal pathway in astrocytes in AD brain (Sasaki et al*.* 2001). The minority of the O-AβpE3-positive structures that is LAMP-1 negative may therefore represent aggregates in the endocytic compartment. It is striking that microglia, the major phagocytosing cells of the brain, do not stain clearly positive for AβpE3 aggregates. An explanation may be that pE-modified Aβ is efficiently degraded in microglia and hence does not accumulate in these cells.

Recently, treatment of transgenic mice with retinoid X receptor (RXR) agonists was shown to enhance clearance of Aβ in an ApoE-dependent manner. The data led the authors to conclude that this was mediated by microglial phagocytosis (Cramer et al. 2012). Whether RXR agonists could also be employed to facilitate clearance of AβpE3 Abeta aggregates by astrocytes and neurons will be interesting to find out. In our neuronal cell culture model, the RXR agonist *trans-*retinoic acid was present in all conditions, and we cannot exclude that this has affected overall uptake to some extent. The exciting study by Cramer et al. again illustrates the high potential of intervention in the uptake and clearance of Aβ as therapeutic strategy for AD. The semiquantitative analysis of the intracellular O-AβpE3 staining in temporal cortex of 16 controls and 11 AD patients showed an age-dependent increase in intracellular staining. Moreover for the intraneuronal O-AβpE3 staining, this age-dependent increase was associated with the disease. This observation underscores the relevance of the oligomeric AβpE peptides in the pathogenesis of the disease. The age-related increase in the intraglial O-AβpE3 staining intensity in control cells might be explained by the resistance of these aggregates against lysosomal degradation. Neuroinflammation is another process that is associated with AD pathogenesis as well as with aging (Giunta et al. 2008) and it will be interesting to investigate whether the inflammatory profile affects the accumulation/clearance of AβpE peptides. A correlation between intraneuronal Aβ_1−*x*_ *staining* and ApoE genotype has been reported (Christensen et al*.* 2010); however, we did not observe any relation between intracellular oligomeric pE Aβ and ApoE genotype in our cohort.

**Fig. 7:**
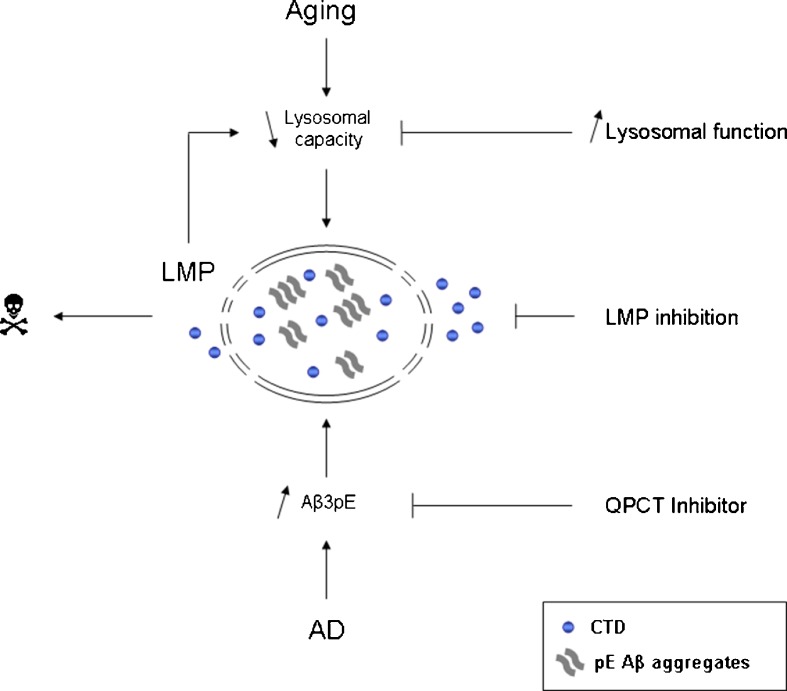
Model showing convergence of aging and Aβ toxicity at the lysosome. Due to an age-related decline in lysosomal activity, the degradation of Aβ is hampered resulting in the accumulation of the degradation-resistant pE variant in the lysosomes. During AD progression, Aβ and QPCT levels increase which leads to further accumulation. The presence of pE Aβ aggregates in the lysosomes causes lysosomal damage. Consequently, the lysosomal degradation capacity will decline even further, which may ultimately contribute to neuronal loss. Preventing Aβ-induced LMP or enhancing the lysosomal degradation capacity may represent promising therapeutic directions for AD

Based on our experiments, we propose the following model (Fig. 7). During aging the lysosomal activity declines (Chondrogianni et al*.* 2002), which hampers the degradation of Aβ and leads to accumulation of the degradation-resistant pE variant in the lysosomes. In the progression of AD, this is further compromised by the rise in Aβ and QPCT levels. The resulting increased load of pE Aβ aggregates in the lysosomes causes lysosomal damage. Consequently, the lysosomal degradation capacity will decline even further, which may ultimately contribute to neuronal loss. A role for lysosomal dysfunction in AD is supported by data from postmortem brain material: neurons in vulnerable brain regions in AD patients show an increased number of structurally abnormal endosomes, lysosomes, and autophagosomes as well as increased expression of lysosomal hydrolases (Nixon 2007). The convergence of aging and Aβ pathology via decline of the lysosomal pathway is supported by a recently developed conditional *Drosophila* model linking Alzheimer’s neurodegeneration with aging (Ling and Salvaterra 2011). Interestingly, recent evidence indicates that familial AD (FAD) mutations in presenilin 1 (PS1) cause lysosomal impairment (Lee et al*.* 2010). In this respect, it is striking that the intraneuronal pE Aβ staining in PS1 FAD patients is increased compared to sporadic patients (Wirths et al*.* 2010). This again points to an important role for the lysosome in intracellular pE Aβ accumulation. In line with our data and model, a recent report demonstrated that increasing the proteolytic capacity of the lysosome reduces Aβ accumulation in a mouse model (Yang et al*.* 2011). Our results highlight the potential of pE Aβ variants as target for intervention in AD. In addition, preventing pE Aβ-induced LMP or enhancing the lysosomal degradation capacity may indicate a promising therapeutic direction in age-related neurodegeneration.

## Materials and methods

### Preparation of different amyloid-β species

Aβ_1-42_ and Aβ_3-42_ were purchased from Anaspec (San Jose, CA, USA) and Aβ_3(pE)-42_ was purchased from Bachem (Liestal, Switzerland). All Aβ used in this study is synthetic and therefore free of additional unwanted modifications and endotoxin contamination. Preparations were of the highest available grade of purity (>95 % HPLC pure). Aβ oligomers were produced as described before and are ultimately present in aqueous solution buffered at neutral pH (Chafekar et al*.* 2007). High-order aggregates were removed by centrifugation (10 min, 14,000 rpm) at 4 °C. The presence of oligomers was confirmed by electron microscopy and thioflavin T assay.

Aβ peptides containing fluorophore and quencher were synthesized by solid phase synthesis using an Fmoc strategy at the NKI peptide facility. Oregon green 488 (Molecular Probes, Eugene, OR, USA) was introduced at amino acid 22 by covalent coupling of Oregon green 488 iodoacetamide to the cysteine. Quenching of Oregon green fluorescence was achieved with a dabcyl group that had been introduced in the peptide at amino acid 12 by introduction of Fmoc-L-Lys(Dabcyl)-OH. Peptides were purified by RP-HPLC and evidenced the expected molecular mass as determined by mass spectrometry.

### Generation of the O-AβpE3 antibody

To generate a polyclonal antibody, a chicken was immunized with oligomeric Aβ_3(pE)-42_. First, immunization was performed intradermally with 100 μg antigen in Freund's complete adjuvant. Subsequently three boosts were performed with 50 μg antigen in Freund's incomplete adjuvant. Eggs were collected and IgY was purified by precipitation with ammonium sulfate and dissolved in TBS with 0.02 % sodium azide.

### Cell culture and treatment

SK-N-SH and HeLa cells were cultured in Dulbecco’s modified Eagle’s medium with GlutaMAX (Gibco BRL, Carlsbad, CA, USA) supplemented with 10 % fetal calf serum (FCS, Gibco BRL), 100 μg/ml streptomycin, and 100 U/ml penicillin. Cell lines are routinely checked for mycoplasma contamination using a specific 16S rRNA PCR-based assay; all cells used in this study were mycoplasma free. SK-N-SH cells were differentiated in culture medium supplemented with all *trans*-retinoic acid (Sigma, St. Louis, MO, USA) at a final concentration of 10 μM for 5 days. Differentiated SK-N-SH cells were treated with 30 μg/μl hydroxychloroquine (Sigma) for 16 h or with 1 μM Aβ_1-42_ or Aβ_3(pE)-42_ oligomers for 16 h.

For toxicity experiments, HeLa cells were plated (1 × 10^4^ cells/well in a 96-well plate) and transfected with Hsp70_pcDNA3 (coding sequence of human Hsp70 subcloned into the HindIII and BamHI restriction sites of pcDNA3, kindly provided by Eric Reits, AMC, The Netherlands) using Lipofectamine 2000 (Invitrogen Life Technologies, Carlsbad, CA, USA) according to the manufacturer’s protocol. Twenty-four hours after transfection, HeLa cells were treated with 2 μM Aβ_3(pE)-42_ oligomers for another 24 h. Cell viability was estimated by MTT metabolism as previously described (Chafekar et al*.* 2007). Aβ treatments were performed in culture medium without phenol red.

### Dotblot

Aliquots of peptide oligomerization reactions were applied onto 0.1 μm nitrocellulose membranes (Schleicher and Schuell, Keene, NH, USA), incubated with antibodies (Table 2), and analyzed as previously described (Nijholt et al*.* 2011).

**Table 2:** Primary antibodies and their sources and use

Antibody	Species	Mono/polyclonal	Dilution	Company
Antibodies for dot blot and Western blot analysis				
6E10	Mouse	Monoclonal	1:5,000	Covance
O-AβpE3	Chicken	Polyclonal	1:200	Hycult Biotech
2-48	Mouse	Monoclonal	1:5,000	Thomas Bayer
Aβ35-42	Mouse	Monoclonal	1:2,000	Hycult Biotech
Aβ38-42	Mouse	Monoclonal	1:2,000	Hycult Biotech
Cathepsin D	Goat	Polyclonal	1:500	R&D systems
Calnexin	Rabbit	Polyclonal	1:1,000	Cell signaling
Actin	Mouse	Monoclonal	1:1,000	Sigma
Antibodies for immunocytochemistry				
LAMP-1	Mouse	Monoclonal	1:200	Santa Cruz
Cathepsin D	Goat	Polyclonal	1:100	R&D systems
Antibodies for immunohistochemistry				
GFAP	Mouse	Monoclonal	1:10	Momosan
O-AβpE3	Chicken	Polyclonal	1:3,200	Hycult Biotech
CR3/43	Mouse	Monoclonal	1:200	Dako
LAMP-1	Mouse	Monoclonal	1:100	Santa Cruz

Listed are antibody, species, mono- or polyclonal, dilution, and company

### Subcellular fractionation and Western blotting

After treatment, the cells were rinsed with ice cold phosphate-buffered saline (PBS) and subcellular fractionation was performed as described previously (Scheper et al. 2004). Equal amounts of proteins were loaded in 1× SDS sample buffer on 10 % polyacrylamide gels. Afterwards, the proteins were blotted on PVDF membranes (Millipore, Billerica, MA, USA**)** and further processed as previously described (Nijholt et al*.* 2011). The primary antibodies and their dilution factors that were used in this study are listed in Table 2.

### Immunocytochemistry

Immunohistochemistry on differentiated SK-N-SH cells was performed as described previously (Nijholt et al*.* 2011). The primary antibodies and their dilution factors that were used in this study are listed in Table 2. Imaging was performed by confocal microscopy using a Leica TCS-SP2 mounted on an inverted microscope.

### Degradation assay

Immediately prior to use, HFIP-treated peptide films of fluorescently labeled internally quenched Aβ were resuspended in DMSO and further diluted in PBS. Ten picomoles of Aβ (pre-aggregated or not) was added to 5 mU of recombinant CTD (Sigma) in 100 mM sodium acetate pH 5.0. Degradation of quenched peptides was followed by monitoring the increase in fluorescence (excitation, 485–12 nm; emission, 520 nm, Fluostar Omega) at 37 °C. Background fluorescence of peptide incubated in buffer without CTD controls was subtracted. The results were statistically analyzed by Student’s *t* test.

### Immunohistochemistry

Human brain specimens of probable AD and non-demented control cases were obtained at autopsy with a short postmortem interval (The Netherlands Brain Bank, Amsterdam, The Netherlands). Informed consent is available for each patient. Clinical diagnosis, gender, age, PMD, Braak stage, and ApoE of all cases used in this study are listed in Table 1. For immunohistochemical staining, formalin-fixed paraffin-embedded tissue and frozen tissue from the mid-temporal cortex was used. Sections (5-μm thick) were mounted on Superfrost plus tissue slides (Menzel-Gläser, Braunschweig, Germany) and dried overnight at 37 °C. For all stainings on the paraffin-embedded tissue, sections were deparaffinized and subsequently immersed in 0.3 % H_2_O_2_ in methanol for 30 min to quench endogenous peroxidase activity. Either no further treatment was applied, or antigen retrieval was performed by boiling sections for 10 min in a microwave with 10 mM sodium citrate buffer pH 6.0 or by incubating sections for 15 min in formic acid. For the stainings on frozen tissue, the sections were treated for 10 min with acetone. Normal sera and antibodies were dissolved in PBS containing 1 % (*w*/*v*) bovine serum albumin (BSA, Boehringer, Mannheim, Germany). Primary antibodies and their sources are listed in Table 2. Negative controls for all single and double immunostainings were generated by omission of primary antibodies.

For detection of O-AβpE3, sections were preincubated for 10 min with normal goat serum (1:10 dilution; DAKO). O-AβpE3 (see Table 2) was incubated overnight at 4 °C. After washing with PBS, sections were incubated for 30 min with biotin-conjugated goat anti-chicken (1:200 dilution, Vector Laboratories, Burlingame, CA, USA). Subsequently, sections were incubated with Vectastain ABC reagent (1:100, Vector Laboratories) for 60 min. Color was developed using 3,3′-diaminobenzidine (EnVision Detection system/HRP, 1:50 dilution, 10 min; DAKO) as chromogen. Sections were counterstained with hematoxylin and mounted using Depex (BDH Laboratories Supplies, East Grinstead, UK).

Due to the different intracellular O-AβpE3 staining patterns in neurons and glial cells, it was decided to score them separately. Scoring was done on the paraffin-embedded tissues heated in sodium citrate buffer before staining by two independent observers blinded to patient data. For each case, the complete gray matter of the section (± 1 cm^2^) was analyzed. The discrimination between neurons and glial cells was based on morphological criteria, e.g., the size of the nucleus and the presence of a clear nucleolus in the nucleus of the neurons. Moreover, as described in the “Results” section, both cell types display a different staining pattern with the O-AβpE3 antibody. For the quantification of the intraneuronal staining, the number of positive cells and the staining intensity was taken into account. Level 1 was assigned to cases showing no O-AβpE3 staining, level 2 was assigned to cases showing one to five cells with low intensity staining per field using a × 40 objective (0.16 mm^2^), level 3 was assigned to cases showing one to five cells with high intensity staining as well as to cases showing >5 cells with low intensity staining per field, and level 4 was assigned to cases showing >5 cells with high intensity staining per field. The intraglial staining did not show great variation in staining intensity so only the number of positive cells was taken into account. Level 1 was assigned to cases showing no O-AβpE3 staining, level 2 was assigned to cases with <5 positive cells per field, level 3 was assigned to cases with 5–10 positive cells per field, and level 4 was assigned to cases showing >10 positive cells per field. Nonparametric correlation analysis (bivariate and partial) on the intracellular staining of O-AβpE3 antibody was done using the SPSS software.

### Double fluorescent immunohistochemistry

To determine co-localization of O-AβpE3 with GFAP, CR3/43, or with LAMP-1 (see Table 2), sections were preincubated for 10 min with normal goat serum (1:10 dilution, DAKO). Subsequently sections were incubated overnight at 4 °C with a mixture of primary antibodies. After washing with PBS, sections were incubated for 60 min with a mixture of biotin-conjugated goat anti-chicken (1:200 dilution, Vector Laboratories) and ALEXA594-conjugated goat anti-mouse (1:400 dilution, Molecular Probes). To visualize the sections stained with LAMP-1, a Cy3-conjugated donkey anti-mouse (1:200 dilution, Jackson Immuno Research, Westgrove, PA, USA) was used. After washing with PBS, sections were incubated for 60 min with streptavidin-ALEXA488 (1:750 dilution, Molecular Probes). Autofluorescence was quenched using the lipid dye Sudan Black B. Sections were mounted with Aqua Poly/Mount (Polysciences). Imaging was performed by confocal microscopy using a Leica TCS-SP2 mounted on an inverted microscope.

## Electronic supplementary material

Below is the link to the electronic supplementary material.

Online Resource 1 (DOC 255 kb)

## Abbreviations

AD: Alzheimer’s disease
Aβ: Amyloid beta
pE: Pyroglutamate
QC: Glutaminyl cyclase
LMP: Lysosomal membrane permeabilization
