# Supplementary material for: Intracellular accumulation of aggregated pyroglutamate amyloid beta: convergence of aging and Aβ pathology at the lysosome
Source: Age (Dordr). 2012 Apr 4;35(3):673–87. doi: 10.1007/s11357-012-9403-0 (PMC3636379; doi:10.1007/s11357-012-9403-0)

Title:

Intracellular accumulation of aggregated pyroglutamate amyloid beta: Convergence of aging and Aβ pathology at the lysosome.

Authors:

*Line De Kimpe, Elise S. Van Haastert, Archontia Kaminari, Rob Zwart, Helma Rutjes, Jeroen J. M. Hoozemans and Wiep Scheper*

Journal:

Age

Corresponding author:

Wiep Scheper, Department of Genome Analysis, Academic Medical Center, Amsterdam

Email: w.scheper@amc.uva.nl

**Fig. S1** The O-AβpE3 antibody recognizes a conformation specific epitope that is enriched in early intermediates of Aβ_3(pE)-42_ aggregation. (a) Decreasing amounts (1; 0.5; 0.25; 0.125 µg) of oligomers of Aβ_1-42_, Aβ_3-42_ and Aβ_3(pE)-42_ were applied to a nitrocellulose membrane and probed with the O-AβpE3 antibody or with 6E10. The O-AβpE3 antibody detects a conformation specific epitope present in oligomers of Aβ_3(pE)-42_, but not in oligomers of Aβ_3-42_ or Aβ_1-42_ (b) Non-aggregated forms of Aβ_1-42_ and Aβ_3(pE)-42_ were run on a 16% tris tricine gel. Western blots were incubated with O-AβpE3 antibody, 6E10 and a pan Aβ_3(pE)-42_ antibody, 2-48 as indicated. The O-AβpE3 antibody does not detect monomers of Aβ_3(pE)-42_ (c) An Aβ_3(pE)-42_ peptide film was dissolved in DMSO, and the oligomerization reactions were initiated as described in the “Materials and Methods” section. At various time points during the 24 hrs aggregation samples of a 50 µM Aβ_3(pE)-42_ solution were taken for dot blot analysis with the O-AβpE3 antibody and with a pan Aβ_3(pE)-42_ antibody, 2-48. The intensity of the spots was quantified and plotted in time as a ratio of the total amount of Aβ_3(pE)-42_. The signal at time point zero was set to 1. At the start of the oligomerization reaction the intensity of the spots increased, then peaked and slowly decreased again. The increase in the first phase indicates that the conformational epitope is formed during the oligomerization reaction. The subsequent decrease in intensity suggests that the structure of the later aggregates is different from the earlier aggregates or that the epitope becomes less exposed or accessible in the later aggregates. The experiment was repeated three times and a representative experiment is shown.


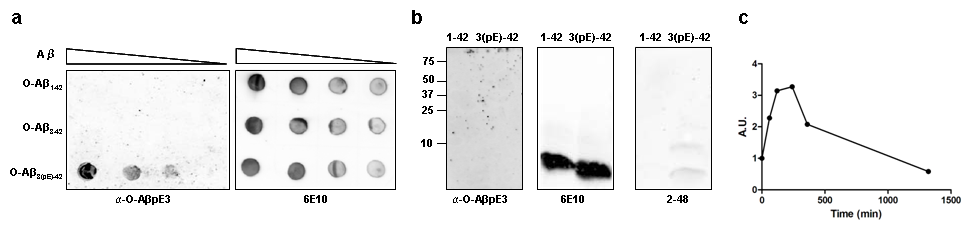


**Fig. S2** Aβ_3(pE)-42_ and Aβ_1-42_ oligomeric aggregates have different structural properties. (a) Decreasing amounts (1; 0.5; 0.25; 0.125 µg) of oligomers of Aβ_1-42_ and Aβ_3(pE)-42_ were applied to a nitrocellulose membrane and probed with the α-Aβ_35-42_ or with α-Aβ_33-38_ antibodies. This analysis showed that the α-Aβ_35-42_ detects both types of oligomers whereas the α-Aβ_33-38_ only binds oligomers of Aβ_1-42_. The 33-38 epitope seems to be differently accessible in both types of oligomers, suggesting a different conformation of the Aβ_1-42_ and Aβ_3(pE)-42_ aggregates. (b) Negative stain transmission electron microscopy (TEM) was used to analyze the global morphology of Aβ_1-42_ and Aβ_3(pE)-42_ oligomers. The Aβ_3(pE)-42_ oligomers are larger than the oligomers of Aβ_1-42_. Scale bar: 200 nm. (c) 50 μM Aβ was aggregated for 24 hrs at different molar ratios of Aβ_3(pE)-42_: Aβ_1-42_, as indicated, applied to nitrocellulose membrane and probed with the O-AβpE3 antibody, with 6E10 or with the 2-48 antibody. The O-AβpE3 did not detect aggregates that consist of a 9-1 molar ratio of Aβ_1-42_ to Aβ_3(pE)-42_. Immunoreactivity was observed using co-aggregates of equimolar amounts of Aβ_1-42_ and Aβ_3(pE)-42_ and further increased with aggregates that consist of higher molar ratios of Aβ_3(pE)-42_ to Aβ_1-42_. These data indicate that co-aggregates of Aβ_1-42_ and Aβ_3(pE)-42_ have a different conformation than the oligomeric aggregates formed by Aβ_3(pE)-42_ alone.


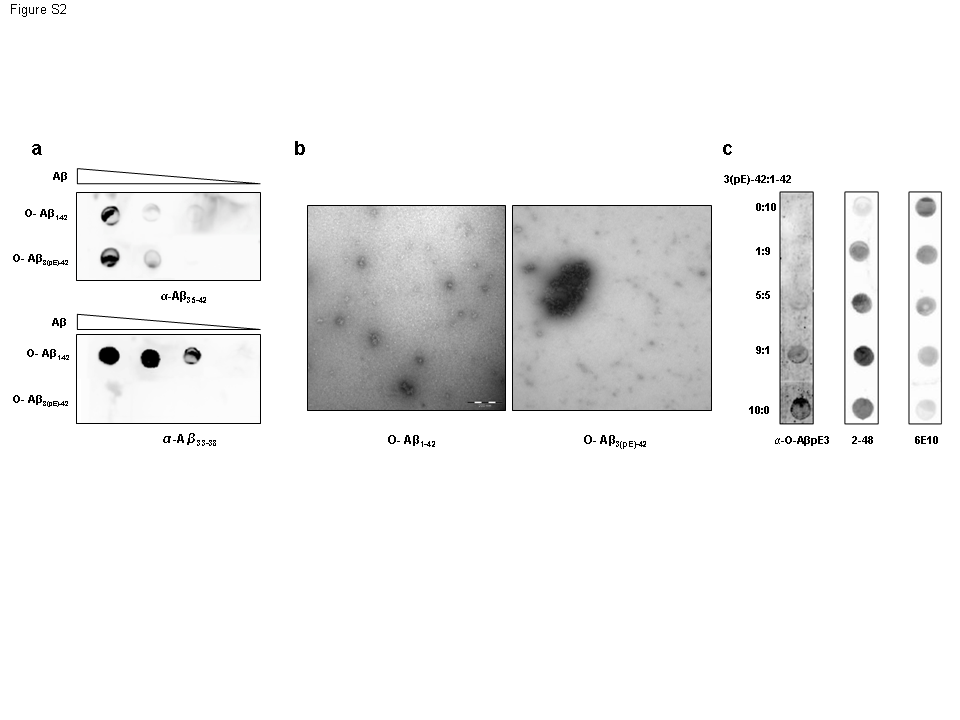

Supplement: Supplementary file 1 — (DOC 255 kb) [file 11357_2012_9403_MOESM1_ESM.doc]
